# *Nfkb2* deficiency and its impact on plasma cells and immunoglobulin expression in murine small intestinal mucosa

**DOI:** 10.1152/ajpgi.00037.2022

**Published:** 2022-08-02

**Authors:** Stamatia Papoutsopoulou, Joseph Tang, Ahmed H. Elramli, Jonathan M. Williams, Nitika Gupta, Felix I. Ikuomola, Raheleh Sheibani-Tezerji, Mohammad T. Alam, Juan R. Hernández-Fernaud, Jorge H. Caamaño, Chris S. Probert, Werner Muller, Carrie A. Duckworth, D. Mark Pritchard

**Affiliations:** ^1^Institute of Systems, Molecular and Integrative Biology, Faculty of Health & Life Sciences, University of Liverpool, Liverpool, United Kingdom; ^2^Department of Biochemistry and Biotechnology, School of Health Sciences, University of Thessaly, Larissa, Greece; ^3^Department of Basic Medical Sciences, Faculty of Dentistry, University of Benghazi, Benghazi, Libya; ^4^Pathobiology and Population Sciences, The Royal Veterinary College, Hatfield, United Kingdom; ^5^Institute of Clinical Molecular Biology, Christian-Albrechts University of Kiel, Kiel, Germany; ^6^Warwick Medical School, Bioinformatics RTP, University of Warwick, Coventry, United Kingdom; ^7^Department of Biology, College of Science, United Arab Emirates University, Al Ain, United Arab Emirates; ^8^Unidad de Investigación, Hospital Universitario de Canarias, Instituto de Tecnologías Biomédicas, La Laguna, Spain; ^9^College of Medical and Dental Sciences, University of Birmingham, Birmingham, United Kingdom; ^10^Lydia Becker Institute of Immunology and Inflammation, Faculty of Biology, Medicine and Health, University of Manchester, Manchester, United Kingdom

**Keywords:** intestinal mucosa, immunoglobulins, plasma cells, NF-κB, Nfkb2, RelB

## Abstract

The alternative (noncanonical) nuclear factor-κB (NF-κB) signaling pathway predominantly regulates the function of the p52/RelB heterodimer. Germline *Nfkb*2 deficiency in mice leads to loss of p100/p52 protein and offers protection against a variety of gastrointestinal conditions, including azoxymethane/dextran sulfate sodium (DSS)-induced colitis-associated cancer and lipopolysaccharide (LPS)-induced small intestinal epithelial apoptosis. However, the common underlying protective mechanisms have not yet been fully elucidated. We applied high-throughput RNA-Seq and proteomic analyses to characterize the transcriptional and protein signatures of the small intestinal mucosa of naïve adult *Nfkb2*^−/−^ mice. Those data were validated by immunohistochemistry and quantitative ELISA using both small intestinal tissue lysates and serum. We identified a B-lymphocyte defect as a major transcriptional signature in the small intestinal mucosa and immunoglobulin A as the most downregulated protein by proteomic analysis in *Nfkb2*^−/−^ mice. Small intestinal immunoglobulins were dramatically dysregulated, with undetectable levels of immunoglobulin A and greatly increased amounts of immunoglobulin M being detected. The numbers of IgA-producing, cluster of differentiation (CD)138-positive plasma cells were also reduced in the lamina propria of the small intestinal villi of *Nfkb2*^−/−^ mice. This phenotype was even more striking in the small intestinal mucosa of *RelB*^−/−^ mice, although these mice were equally sensitive to LPS-induced intestinal apoptosis as their *RelB*^+/+^ wild-type counterparts. NF-κB2/p52 deficiency confers resistance to LPS-induced small intestinal apoptosis and also appears to regulate the plasma cell population and immunoglobulin levels within the gut.

**NEW & NOTEWORTHY** Novel transcriptomic analysis of murine proximal intestinal mucosa revealed an unexpected B cell signature in *Nfkb2*^−/−^ mice. In-depth analysis revealed a defect in the CD38+ B cell population and a gut-specific dysregulation of immunoglobulin levels.

## Graphical abstract

**Figure:**
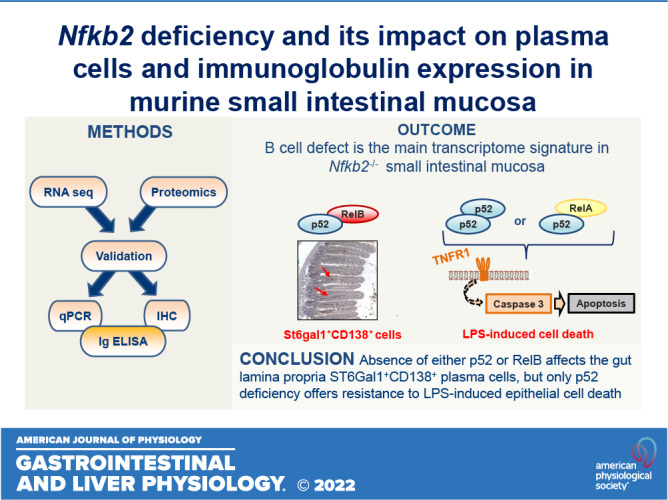

## INTRODUCTION

The NF-κB family of transcription factors consists of ubiquitously expressed proteins that form homo- or heterodimers and drive the transcription of a plethora of genes that in turn regulate the development of the immune system, innate and adaptive immune responses, inflammation, and cancer (1–5). NF-κB activation occurs via two major signaling pathways, the canonical and the noncanonical (6, 7). Activation of the noncanonical (alternative) NF-κB pathway is under tight control, and the central signaling component is NF-κB-inducing kinase (NIK; also known as MAP3K14), which induces p100 phosphorylation via activation of the inhibitor of NF-κB kinase α (IKKα) (8–11). This leads to p100 processing and to a generation of the p52 subunit and the translocation predominantly of the p52/RelB heterodimer to the nucleus (6). Typical inducers of this pathway are members of the tumor necrosis factor receptor (TNFR) superfamily. Most of these receptors also stimulate the canonical NF-κB pathway and mediate biological processes that involve functional cooperation between the two NF-κB pathways (12).

Work on genetically modified mice has shown that the p52/RelB dimer regulates the development of secondary lymphoid tissues, such as the spleen, lymph nodes, and Peyer’s patches (13, 14). Similarly, p100/p52 has been shown to be important for the maintenance of the peripheral B cell population and humoral responses in mice (15, 16). In humans, loss of NIK leads to severe immune defects, whereas the overexpression of NIK is observed in inflammatory diseases, metabolic disorders, and during the development and progression of cancer (17). Similarly, some patients with immunodeficiencies have been shown to have point mutations in the *NFKB2* gene (18–21).

The role of the noncanonical NF-κB pathway in the gut has also been studied, mainly using transgenic mouse models. Defects in noncanonical NF-κB signaling render mice more sensitive to the intestinal pathogen *Citrobacter rodentium,* leading to gut inflammation, whereas enhanced activation due to defects in upstream inhibitors promotes host defense against infections (22). NIK signaling has also been shown to regulate dendritic cell function and protect against *Citrobacter rodentium* infection (23). In contrast, constitutive NIK activation has been demonstrated to be associated with colitis in mice and in patients with ulcerative colitis (24). *Nfkb2*^−/−^ mice showed resistance to dextran sulfate sodium (DSS)-induced colitis and azoxymethane (AOM)/DSS-induced colitis-associated colon cancer (25). This strain was also resistant to lipopolysaccharide (LPS) and TNF-induced small intestinal apoptosis and cell shedding in vivo (26, 27)_._ Infection studies have also shown that *Nfkb2^−/^*^−^ mice have a reduced ability to clear the gut helminth *Trichuris muris* (28) and the gastric pathogen *Helicobacter felis*, the latter observation being associated with reduced gastric preneoplastic pathology due to a defective immune response (25). Moreover, small intestinal enteroid cultures from *Nfkb2*^−/−^ mice were shown to be more resistant to TNF-induced death, compared with wild-type controls, as revealed by maintained morphology and detailed immunohistochemical approaches (29). The resistance that p100/p52 deficiency offers to gastrointestinal tissues during various in vivo inflammatory models is therefore striking and strongly suggests the presence of an underlying common factor.

In this study, we used RNA-sequencing and proteomics analyses on small intestinal mucosae to characterize tissue-specific expression profiles in naive adult *Nfkb2*^−/−^ mice. The transcriptional signature identified B cell-specific defects. Proteomics analysis and further validation experiments demonstrated a considerable reduction of IgA levels and of cluster of differentiation (CD)138-positive (CD138+) plasma cells in the lamina propria of the *Nfkb2*^−/−^ small intestine. Similar and even more pronounced defects in plasma cells were identified in *RelB*^−/−^ small intestine, but this genotype did not show the same protection against LPS-induced apoptosis that has previously been described in *Nfkb2*^−/−^ mice.

## MATERIALS AND METHODS

### Mice

Wild-type (WT) C57BL/6 mice supplied by Charles River (Margate, UK) and transgenic strains on the same C57BL/6J background, including *Nfkb2^−/−^* (15) and *RelB*^−/−^ (30) mice (and their wild-type and heterozygous counterparts), were maintained at the University of Liverpool. Same-sex litter mates were housed together in individually ventilated cages with up to five mice per cage. All mice were maintained on a regular diurnal lighting cycle (12:12 light:dark) with ad libitum access to food (irradiated CRM(P), SDS Special Diet Services) and water. The cage substrate was “Lignocel select,” a poplar granulate (IPS, Ltd., International Product Supplies UK). Environmental enrichment included nesting material Z/Nest (IPS, Ltd.), PVC pipe, and shelter. Mice were housed under specific pathogen-free conditions in the Biomedical Services Facility of the University of Liverpool, accredited by the UK Home Office and operating under UK legislation (ASPA, Animals Scientific Procedures Act 1986). All procedures were performed on adult mice (minimum age: 8 wk) under appropriate UK Home Office licenses. LPS from *Escherichia coli* O111:B4 purified by phenol-extraction (Sigma Aldrich, Gillingham, UK) was diluted in sterile phosphate-buffered saline (PBS). Low-dose LPS (0.125 mg/kg body wt) was administered to mice by intraperitoneal injection as described previously (27), and the mice were euthanized 1.5 h later. All mice were euthanized by CO_2_ administration, confirmed by cervical dislocation, with ethical approval under UK Home Office legislation (Animals Scientific Procedures Act 1986) and local ethical approval.

### Genotyping

Genomic DNA was extracted from ear clips using a modified HotSHOT protocol (31). PCR was performed in 1X GoTaq Green master mix (Promega) containing 2.5 µM of each forward and reverse primers. For *Nfkb2* genotyping the primers used were the following: *1*) Forward Common: 5′-GCCTGGATGGCATCCCCG-3′, *2*) Reverse WT: 5′-GTTTGGGCTGTTCCACAA-3,′ and *3*) Knockout *Nfkb2:* 5′- CCAGACTGCCTTGGGAAA-3′. The PCR conditions were the following: 1 cycle of 60 s at 95°C, 35 cycles of 60 s at 95°C, 90 s at 55°C, and 30 s at 72°C, and extension for 120 s at 72°C. Wild-type *Nfkb2* alleles generated a 230-bp PCR product, and the disrupted *Nfkb2* alleles generated a 120 bp product. For *RelB* genotyping the primers for the WT allele were the following: Forward 5′- GTGGTGCCCGGGAATAGGATTGCT-′3 and Reverse 5′- CCATTTTGCTCTGGGTCTGTGTCT G-3′ and for the targeted allele were the following: Forward 5′- CATCGACGAATACATTAAGGAGAACGG-3′ and Reverse 5′- AAATGTGTCAGTTTCATAGCCTGAAGAACG-3′. The PCR conditions were the following: 1 cycle of 120 s at 95°C, 35 cycles of 60 s at 95°C, 90 s at 60°C, and 60 s at 72°C, and extension for 120 s at 72°C. Wild-type *Nfkb2* alleles generated a 375-bp PCR product, and the disrupted *RelB* alleles generated a 225 bp product. The PCR products were then analyzed by electrophoresis in a 2% (wt/vol) agarose gel with TAE (Tris-Acetate-EDTA, pH 8.0) buffer.

### RNA Sequencing, Read Mapping, and Analysis of Differential Expression

C57BL/6 or transgenic male mice (*n* = 6), between 8 and 10 wk old, were euthanized by CO_2_ administration. The proximal small intestine was dissected and rinsed with phosphate-buffered saline (PBS), and mucosal scrapes were used for isolation of total RNA using the RNeasy Mini Kit (QIAGEN). RNA was prepared as previously described (32), with RNA integrity assessed using an Agilent Bioanalyzer following the manufacturer’s instructions. Strand-specific sequencing libraries were prepared with the TruSeq stranded Total RNA kit (Illumina) from 1 µg total RNA of each sample and sequenced on an Illumina HiSeq2000 (100-nucleotide paired-end reads). Raw reads passing the chastity filter from Illumina were first preprocessed using cutadapt (33) and PrinSeq-lite (34) to remove adapter and low-quality sequences. The reads were aligned to nonrepeat masked version of the *Mus musculus* reference genome (GRCm38) using TopHat2 (35), whereas the corresponding GTF annotation file was obtained from the Ensembl database (Mus_musculus.GRCm38.80.gtf). The DESeq2 package from BioConductor was used for differential expression analysis (36). DESeq2 is based on a model using the negative binomial distribution and uses a normalization procedure based on sequencing depth and biological variance. Log2-fold changes (between transgenic and wild-type control) and adjusted *P* values (corrected for multiple testing using the Benjamini and Hochberg method) were calculated and genes were selected as differentially expressed with an absolute fold-change ≥ 2 and adjusted *P* value of < 0.05. Gene ontology enrichment analysis was performed using the GOnet online tool (37).

### Proteomics Analysis of Intestinal Tissue

Female naive C57BL/6 and *Nfkb2^−/−^* mice (*n* = 5/genotype), 9–11 wk old were used for the proteomics analysis. Crypts were isolated from the proximal small intestine as described previously (29). Briefly, the intestine was opened longitudinally, cut in small pieces, and washed extensively with cold PBS pH 7.8. The tissue was incubated in buffer containing 0.5 mM EDTA for 30 min at 4°C with shaking. The pieces were let to settle, and the buffer was removed by aspiration and replaced with small volume (1 mL) of hypertonic buffer, followed by extensive shaking/vortexing to help the crypts detach from the mucosa. The isolated crypts were lysed in 250 µL of 0.1 M triethylammonium bicarbonate (TEAB) containing 0.1% sodium dodecyl sulfate (SDS) by incubating for 30 min on ice. The lysate was centrifuged at 13,000 rpm for 15 min at 4°C, and the supernatant was stored at −80°C for further use.

Protein concentration was measured using the Qubit Fluorometer (Invitrogen). Aliquots of 300 μg total protein were precipitated using the methanol-chloroform method (38). The final pellet was air-dried and digested overnight in 50 μL of 50 mM HEPES (pH 8.0), 60 ng/μL of trypsin (Promega), and 20 ng/μL of LysC (Promega). After protein digestion, HEPES concentration was adjusted to 200 mM, and 25 μg of peptides per sample was mixed with 200 μg of TMT10plex labels (Thermo Scientific) in 20 μL acetonitrile and incubated for 2 h at RT. Samples were stored at −20°C until further use. A pool of all samples (1 μg each) was analyzed by liquid chromatography-mass spectrometry (LC-MS) as described previously (39), to check the labeling efficiency and calculate the coefficient of variance. After corrections were loaded, the reaction was quenched with 1 μL of 5% hydroxylamine, and 15 μg of peptides per sample were combined for fractionation using High-pH Reversed-Phase Peptide Fractionation Kit (Pierce, Thermo Fisher). All LC-MS fractions were dried in a vacuum concentrator (Eppendorf) and dissolved in 50 μL of 2% acetonitrile and 0.1% TFA in a sonication bath for 5 min. Peptides were eluted at 250 nL/min by increasing the mobile phase B from 5% B to 22% over 129 min and then 35% B over 27 min, followed by 80% B for 3 min and a 15-min reequilibration at 4% B. MS data was acquired with Xcalibur v3.0.63 (Thermo Scientific). Electrospray used a static Nanospray-Flex with a stainless-steel emitter OD 1/32’ in positive mode at 1.8 kV (Thermo Scientific). MS survey scans from 375 to 1,500 *m/z*, with an 8 × 10^5^ ion count target, maximum injection time of 150 ms, and resolution of 60,000 at 200 *m/z*, acquired in profile mode were performed in the Orbitrap analyzer. Data-dependent mode selected the most abundant precursor ions possible in a 3-s cycle time followed by 60 s exclusion, and ions were isolated in the quadrupole with a 1-m/z window when their intensity was above 40,000. MS/MS scans were performed in the Orbitrap with a 4 × 10^4^ ion count target, maximum injection time of 500 ms, and resolution of 60,000 at 200 m/z, acquired in centroid mode. Precursor ions were fragmented with higher energy C-trap dissociation (HCD), normalized collision energy of 38%, and fixed first mass of 120 m/z. Thermo Scientific raw files were analyzed using MaxQuant software v1.6.0.163 against the UniProtKB Mouse database (57,258 entries, release June 2016). Peptide sequences were assigned to MS/MS spectra as described previously (39). Reporter ion MS2 and 10plex TMT protein quantification were calculated. Data processing was performed using the Perseus module of MaxQuant v1.6.0.16.4 (40). Proteins identified by the reverse, contaminant, and only-by-site hits were discarded. Only protein groups identified with at least two assigned peptides were accepted, data was normalized as previously described (41), and intensities were log2 transformed. Protein groups with significant intensity regulation were determined according to Welch’s *T* test using the Perseus proteomics data analysis tool (42). Significant hits were filtered using permutation-based False Discovery Rate <5%. The data set has been deposited to the ProteomeXchange Consortium (43) via the PRIDE partner repository (44). This work is MIAPE-compliant (45).

### Real-Time PCR

Equal number of male (*n* = 3 or 4) and female (*n* = 3 or 4) naive C57BL/6 and *Nfkb2*^−/−^ mice, 9–11 wk old, were used for validation experiments by real-time PCR. Small intestinal mucosal scrapes were used for total RNA isolation using the RNeasy Mini Kit (QIAGEN). Reverse transcription was performed using the High-Capacity cDNA Reverse Transcription Kit (Applied Biosystems, Thermo Fisher Scientific, UK). cDNA (40 ng) was used as a template for real-time PCR reactions using TaqMan gene expression assays for *Hprt* (Mm00446968_m1), *St6gal1* (Mm00486119_m1), *Cd22* (Mm00515432_m1)*, Gbp2* (Mm00494576_g1), and *Gbp8* (Mm00780575_s1). Real-time PCR was performed on a Roche LightCycler 480, and cycling conditions were performed as per the manufacturer’s instructions. Relative quantification was performed using standard curves based on serial dilutions of samples that had positive signals for the gene of interest.

### Tissue Processing and Immunohistochemistry

Tissues were fixed in 4% paraformaldehyde and embedded in paraffin wax. Sections of 3- to 5-µm thickness were subsequently microtomed and transferred onto charged glass slides for immunohistochemistry. Tissue sections were treated with 1% hydrogen peroxide in methanol to block endogenous peroxidases, followed by heat-induced antigen retrieval in 0.01 M citrate acid buffer (pH 6.0) for 20 min in a 800-W microwave. The primary antibodies used were mouse monoclonal anti-mouse St6gal1 antibody (Cat No. MA5-11900, Invitrogen, Thermo Fisher Scientific, UK, 1:50 dilution), Mouse anti-GBP1-5 (1:100; sc-166960; Santa-Cruz, Heidelberg, Germany), purified anti-mouse CD138 (Syndecan-1) antibody (Cat No. 142502, BioLegend UK LTD, London, UK, 1:400 dilution), and rabbit polyclonal anti-active-caspase-3 antibody (Cat No. AF835, R&D Systems, Abingdon, UK, 1:1,000 dilution). Peroxidase-labeled anti-rabbit EnVision (Dako, Cambridge, UK), Mouse on Mouse (M.O.M.) ImmPRESS HRP (peroxidase) Polymer kit (Vector Laboratories, Peterborough, UK), and 3,3′-diaminobenzidine were used for visualization. Active-caspase-3-positive cells were counted from the base of the villus (above crypt level) to the midpoint of the villus tip in 18–20 well-orientated hemivilli, as described previously (27). The intestinal epithelial cells were characterized as *1*) “apoptotic” if there was defined positive staining that was confined to cytoplasmic or nuclear borders and *2*) “shedding” if there was defined positive staining that was confined to cytoplasmic or nuclear borders and in addition, there was apical elevation of the cytoplasmic membrane, and/or an apically positioned nucleus.

### ELISA

For quantification of immunoglobulins, tissues were dissected and homogenized in ice-cold RIPA buffer (Thermo Fisher Scientific, UK). After 20 min on ice, the lysates were centrifuged for 10 min at 10,000 *g*, and the clear supernatant was transferred to a new Eppendorf tube. For serum samples, blood was withdrawn by cardiac puncture, incubated for 30 min at room temperature, and centrifuged for 10 min at 2,000 *g* at 4°C. The clear supernatant was transferred to a new tube. All samples were stored at −80°C until further use. Immunoglobulin A (IgA) was measured with the IgA Mouse Uncoated ELISA Kit, Immunoglobulin M (IgM) was measured with the IgM Mouse Uncoated ELISA Kit, and all the immunoglobulins were measured with an Ig Isotyping Mouse Uncoated ELISA Kit (all from Invitrogen, Thermo Fisher Scientific, UK).

### Statistical Analysis

Data from the in vitro and in vivo experimental datasets are expressed as means ± standard error of mean (SEM). After assessment for normality and equality of variances, statistical inferences on data were performed using the Mann–Whitney test for pairwise comparisons or Kruskal–Wallis test for multiple comparisons. Differences were considered statistically significant when *P* < 0.05 using StatsDirect v3.0.171 (StatsDirect, Ltd.; Birkenhead, UK).

All authors had access to the study data and have reviewed and approved the final manuscript.

## RESULTS

### *Nfkb*2 Deficiency Alters the Small Intestinal Mucosa Transcriptional Signature in Naïve Mice

**Figure 1.**
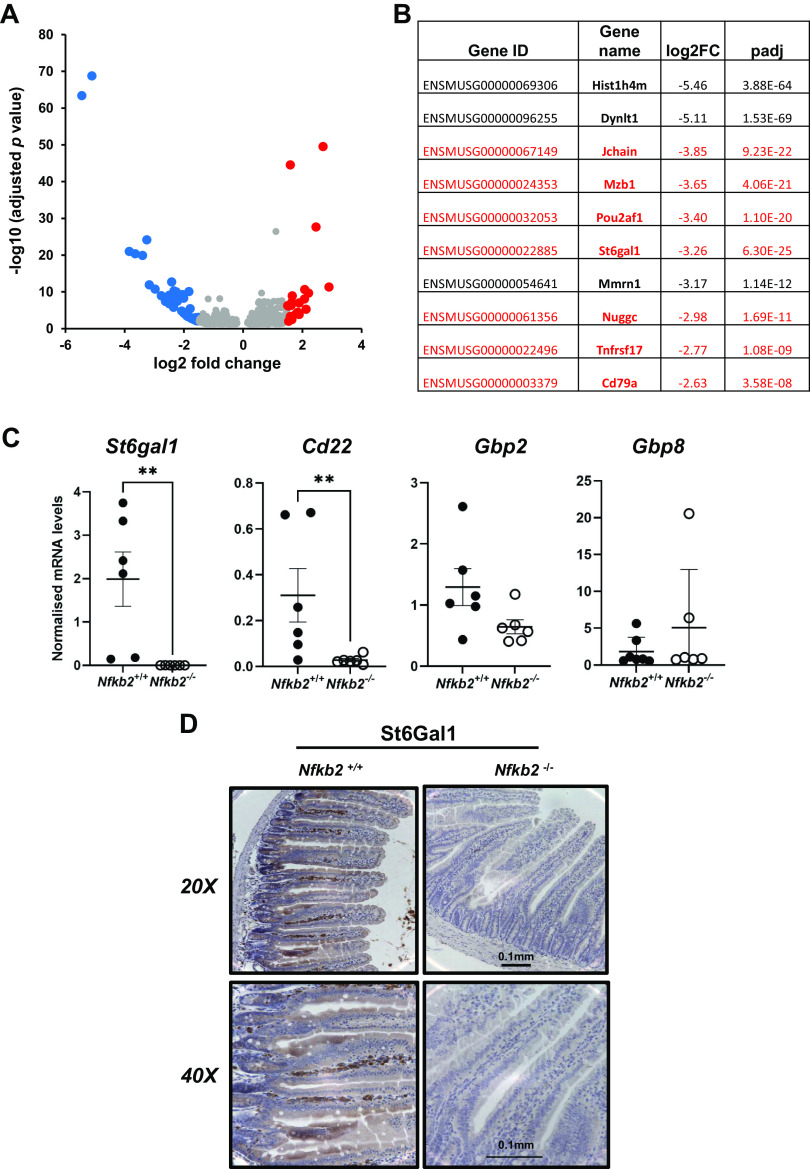
B cell defect is a major transcriptional signature in *Nfkb2*^−/−^ small intestinal mucosa. Total RNA was isolated from proximal small intestine and used for RNA sequencing (*n* = 6). *A*: volcano graph of the differentially regulated genes that were further filtered, under high stringency, using a cut off of 1.5 log2-fold change for both downregulated genes (blue) and upregulated genes (red). *B*: top 10 downregulated genes in *Nfkb2*^−/−^ small intestinal tissue with genes that are expressed in *B* cells shown in red. *C*: validation of *St6gal1, Igtp, Gbp2*, and *Gbp8* gene expression by real-time qPCR analysis in *Nfkb2*^+/+^ (black circles) and *Nfkb2*^−/−^ (open circles) samples (*n* = 6–8/genotype, equal number of males and females). *D*: proximal small intestinal sections showing lamina propria staining for St6gal1 in C57BL/6, but not in *Nfkb2*^−/−^ mice (representative image of *n* = 4). Mann–Whitney test for pairwise comparisons was applied. Differences were considered statistically significant when *P* < 0.05.

RNA-sequencing analysis was performed on total RNA that was extracted from small intestinal mucosal scrapes isolated from age- and sex-matched C57BL/6J and *Nfkb2*^−/−^ mice (*n* = 6 males/group). Informatics analysis revealed a high number of differentially expressed genes in *Nfkb2*^−/−^ mice compared with wild-type small intestine, as visualized by volcano plot (Fig. 1*A*). Five hundred and eighty-seven statistically significant differentially expressed genes were identified, with 352 being upregulated and 235 being downregulated in the absence of *Nfkb2*. For further analysis, a more stringent cut-off filter was applied and the genes demonstrating log2-fold changes of either less than −1.5 or higher than 1.5 only were selected, resulting in the most significantly regulated genes in the small intestine of *Nfkb2*^−/−^ mice (*n* = 69) being used for further analysis (37) (Supplemental Table S1; see https://doi.org/10.6084/m9.figshare.19146497.v1). Gene ontology (GO) enrichment analysis showed that B cell proliferation, humoral immune responses, and interferon-regulated pathways were the main biological processes affected by the absence of *Nfkb2* in the small intestine (Supplemental Table S2). More than 50% of the downregulated genes were shown to be B cell-specific, and seven of these genes were among the top 10 on the gene expression list (Fig. 1*B*). Among the differentially downregulated genes, we identified genes that are known to be responsible for homing of B cells in the gut. These included the *Cd22* gene that encodes the B cell-specific receptor Cd22 (Siglec 2) that binds to α 2,6-linked sialic acid ligands attached to galactose and the *St6gal1* gene, that encodes β-galactoside α-2,6-sialyltransferase 1 (ST6Gal1), the enzyme that catalyzes this specific sialylation reaction (46). Validation at the mRNA level by real-time PCR analysis showed a statistically significant reduction of *St6gal1* and *Cd22* mRNA in small intestinal mucosal scrapes from *Nfkb2*^−/−^ mice compared with C57BL/6 controls (Fig. 1*C*). *Gbp2* and *Gbp8*, that encode members of the guanylate-binding protein family (47) and showed upregulation in the absence of *Nfkb2* were differentially expressed, with only Gbp8 showing higher levels in the *Nfkb2*^−/−^ (Fig. 1*C*). At the protein level, immunohistochemical (IHC) analysis matched the mRNA observations for St6Gal1 that was expressed in cells within the villus lamina propria of C57BL/6 mice, but there was no expression of this protein in the small intestinal mucosa of *Nfkb2*^−/−^ mice (Fig. 1*D*).

### Plasma Cell Deficiency and Dysregulation of Immunoglobulins in *Nfkb2*^−/−^ Small Intestine

**Figure 2.**
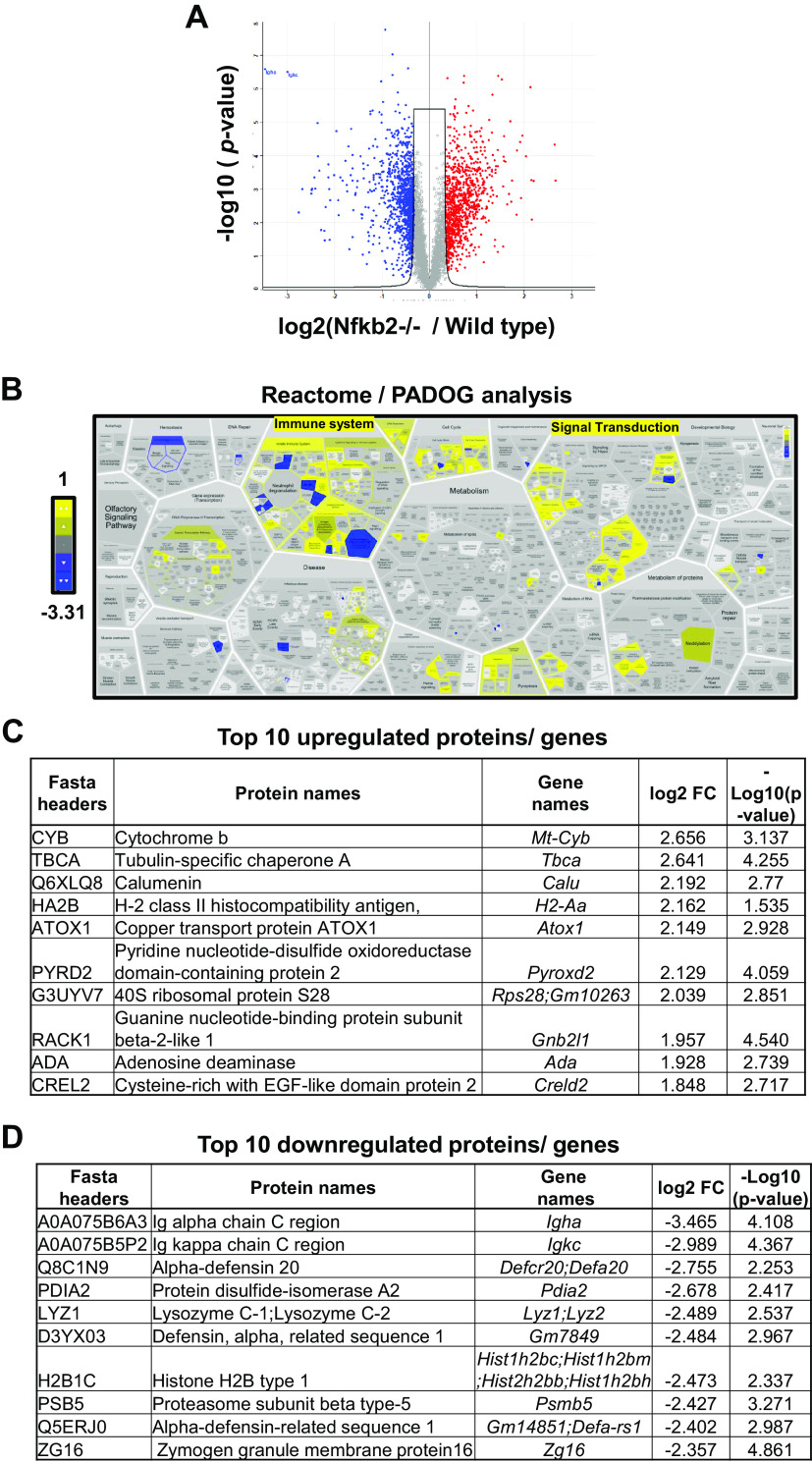
Proteomics analysis of *Nfkb2*^−/−^ small intestinal mucosa reveals immune system category and immunoglobulins as being the most affected. Total protein lysates from proximal small intestinal epithelium of both C57BL/6 wild-type and *Nfkb2*^−/−^ mice (*n* = 4) were used for proteomics analysis. *A*: volcano plot showing the differentially regulated proteins (Welch´s *T* test, FDR < 0.05) for both downregulated (blue) and upregulated proteins (red). *B*: pathway analysis with down-weighting of overlapping genes (PADOG) showing the most downregulated [blue, lowest value log2(fold change) = −3.31] and upregulated [yellow, highest value log2(fold change) = 1] enriched pathways affected by *Nfkb2* deletion. Scale bar represents the log2 (fold change). *C* and *D*: top 10 most upregulated and downregulated proteins identified by proteomics analysis in proximal small intestine isolated from naïve *Nfkb2*^−/−^ mice.

**Figure 3.**
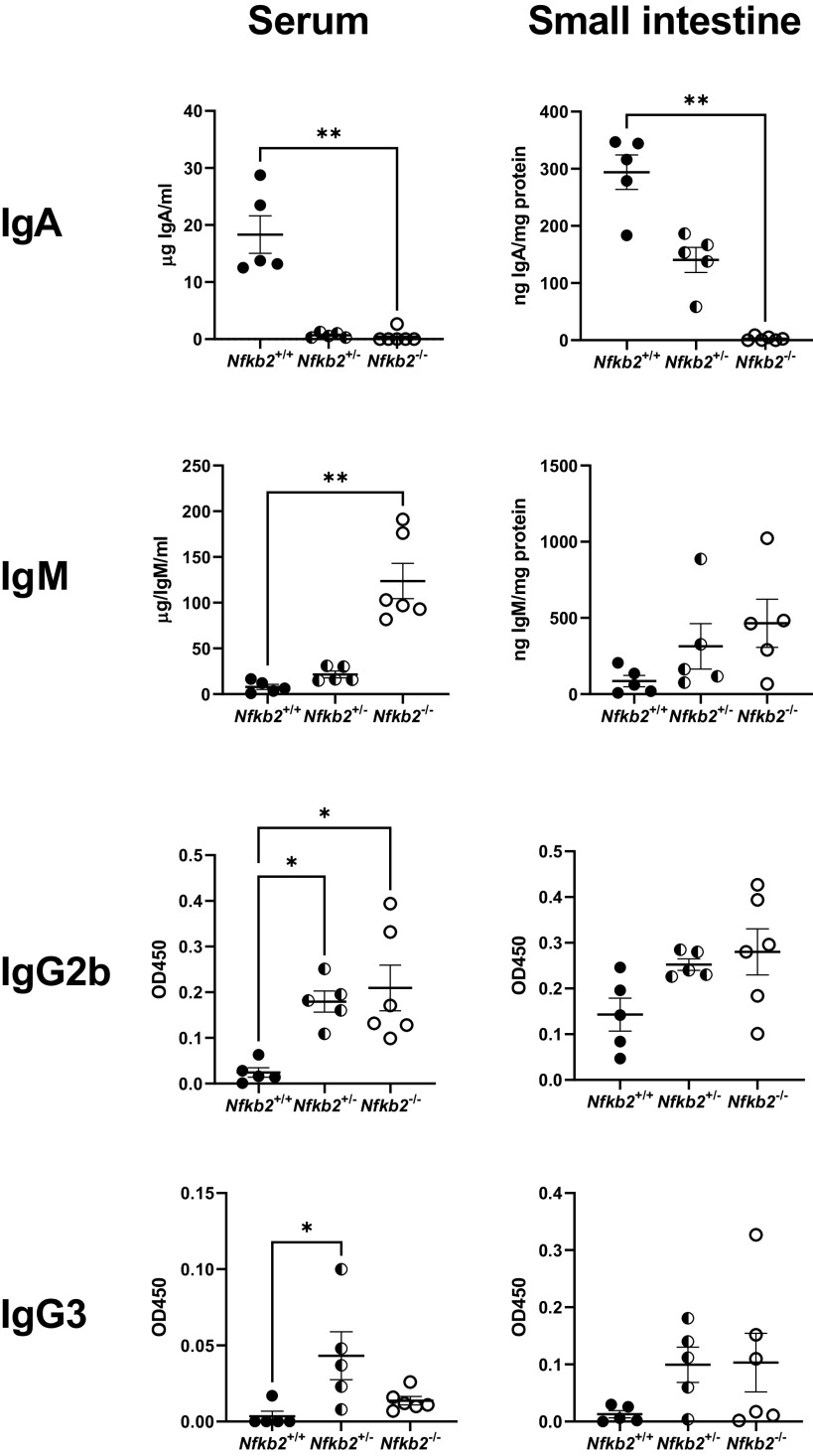
Severe dysregulation of immunoglobulins in *Nfkb2*^−/−^ mice. Small intestinal tissue lysates and sera from *Nfkb2^+/+^* (black circles), *Nfkb2*^+/−^ (black and white circles), and *Nfkb2*^−/−^ mice (open circles) (*n* = 5 or 6/genotype) were used for quantification of immunoglobulins. Total protein in tissue lysates was used for normalization purpose. Kruskal–Wallis multiple-comparison test was applied. *P < 0.05, **P < 0.01.

Small intestinal epithelia from naive C57BL/6 and *Nfkb2*^−/−^ mice (*n* = 5 females/genotype, 9–11 wk old) were lysed, and the lysates were further processed for proteomic analysis. Bioinformatics processing indicated that 1,469 proteins were differentially expressed in the *Nfkb2*^−/−^ tissue compared against C57BL/6 (Fig. 2*A*). A cut-off threshold of FDR ≤ 0.05 was applied, which resulted in 652 upregulated and 820 downregulated proteins. Pathway analysis with down-weighting overlapping genes (PADOG) showed high enrichment of affected (mainly downregulated) proteins in the immune system (Fig. 2*B* and Supplemental Fig. S1). The pathways found differentially expressed at an adjusted *P* value ≤ 0.05 are considered to be significantly regulated. The top 10 most upregulated and downregulated proteins are presented in Fig. 2, *C*and *D*, respectively. Immunoglobulin A (IgA) was identified as the most downregulated protein in the *Nfkb2*^−/−^ samples, and CD138/Syndecan-1 was also among the significantly downregulated proteins. This is a specific marker of plasma cells that are the main producers of IgA in the gut (48). We therefore set to examine the concentrations of IgA in both sera and small intestinal lysates from both *Nfkb2^+/+^* (*n* = 5) and *Nfkb2*^−/−^ mice (*n* = 6). For this purpose, we used *Nfkb2*^−/−^ mice, heterozygous *Nfkb2^+/−^* mice, and *Nfkb2*^+/+^ as wild-type littermate controls. As shown in Fig. 3, the concentrations of IgA were markedly decreased in both the small intestinal lysates and serum of *Nfkb2^+/−^* mice and were undetectable in homozygous *Nfkb2*^−/−^ mice. In contrast, a massive increase in the concentration of IgM was detected in both the serum and small intestinal lysates of these mutant mice. Similarly, we observed higher IgG2b and IgG3 levels in *Nfkb2^+/−^* and *Nfkb2*^−/−^ animals, as reflected by the OD_450_ in the immunophenotyping assays used (Fig. 3). However, IgG1 and IgE were not detected by the assays used.

All the abovementioned data led to the hypothesis that there is an Nfkb2-dependent defect in lamina propria plasma cells, the main producers of IgA in the small intestine. Therefore, we conducted further immunohistochemistry experiments on serial histological sections and showed that St6Gal1 stains terminally differentiated, CD138+ plasma cells, but not general B cell populations that are stained for CD20, a receptor that is expressed by the majority of B cells, but whose expression is lost in terminally differentiated plasma blasts and plasma cells (Supplemental Fig. S2).

**Figure 4.**
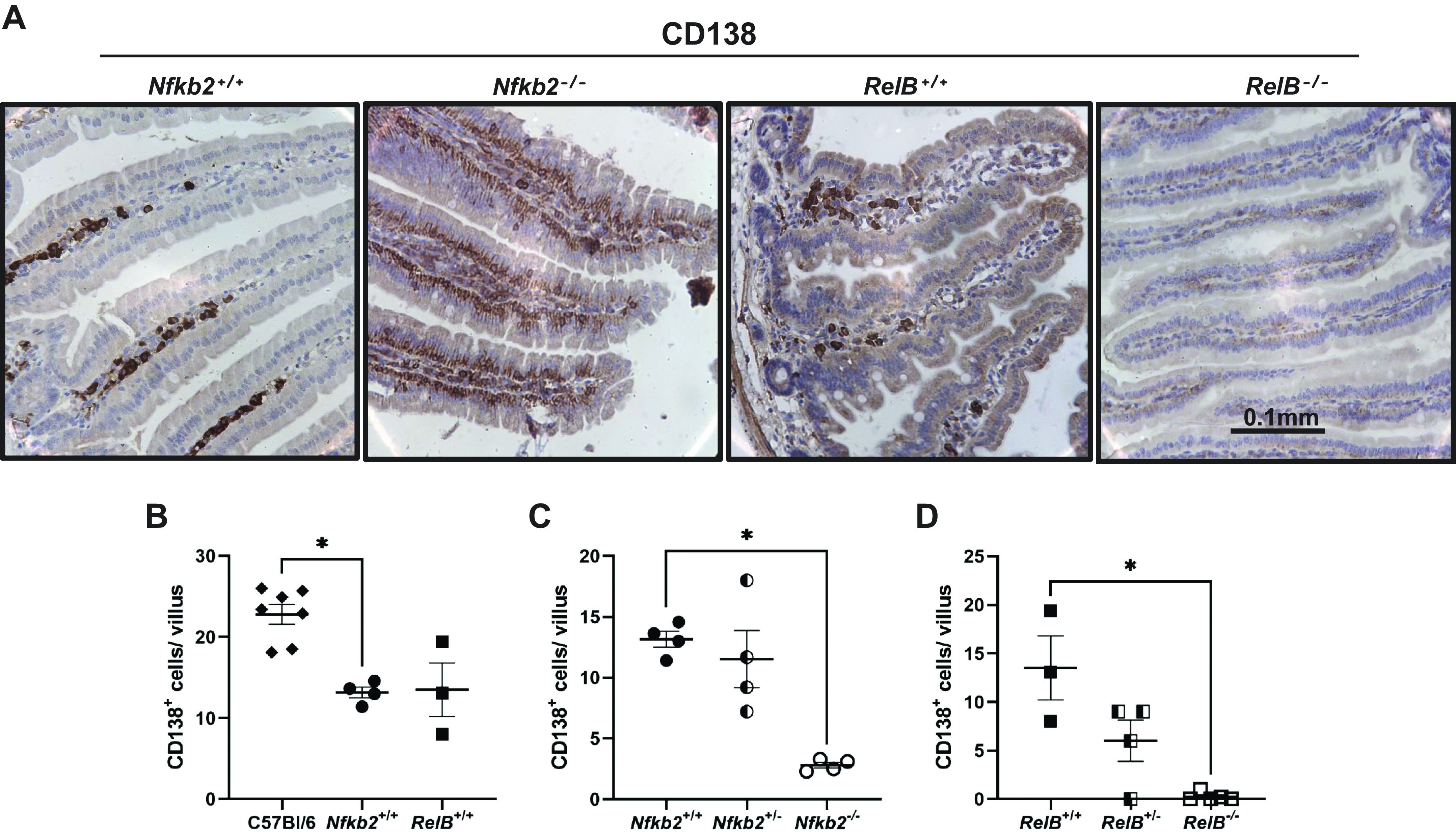
Absence of CD138-positive (CD138+ve) plasma cells in *Nfkb2*^−/−^ and *RelB*^−/−^ intestinal lamina propria. Small intestine from C57BL/6 and *Nfkb2*^−/−^ mice (males and females) and *RelB*^−/−^ females (along with their wild-type and heterozygous littermates) were fixed and 4 µm sections were used for staining for CD138 (×40 magnification). *A*: representative images of stained villi of the four genotypes are presented. Tissues were counterstained with hematoxylin. *B*: comparison of CD138+ve plasma cells numbers between C57BL/6 (black diamonds) and the respective wild-type littermate controls [*Nfkb2^+/+^* (black circles) or *RelB*^+/+^ (black squares)]. *C*: effect of *Nfkb2* deficiency on lamina propria CD138+ve plasma cell numbers in *Nfkb2^+/+^* (black circles), *Nfkb2*^+/−^ (black and white circles), and *Nfkb2*^−/−^ (open circles) samples. *D*: effect of *RelB* deficiency on lamina propria CD138+ve plasma cell numbers in *RelB^+/+^* (black squares), *RelB^+/−^* (black and white squares), and *RelB*^−/−^ (open squares) samples. Ten villi per mouse were counted (*n* = 3–7 mice/genotype). Kruskal–Wallis multiple-comparison test was applied. *P < 0.05. Scale bars represent 100 μm.

Immunohistochemistry for CD138 showed strong staining in C57BL/6 small intestinal lamina propria where B cells reside, as well as some positive staining at the basolateral membrane of intestinal epithelial cells. However, the lamina propria B cell staining was considerably reduced in *Nfkb2*^−/−^ mice (Fig. 4*A*). In this experiment, we also included sections from *RelB*^−/−^ mice, as RelB is the major binding partner of p52 in the active NF-κB heterodimer. *RelB*^−/−^ sections had an even more pronounced phenotype, where there was little CD138 staining in the lamina propria and reduced staining of the epithelial cells of the small intestinal villi (Fig. 4*A*). Quantification of CD138-positive plasma cells showed that C57BL/6 wild-type mice had a small, but statistically significant increase in the number of CD138-positive cells compared with the wild-type littermate controls from the two mutant colonies (*Nfkb2*^+/+^ and *RelB*^+/+^) (Fig. 4*B*). Therefore, we used the wild-type littermate controls for further comparisons. As shown in Fig. 4*C*, there was a substantial reduction in the number of CD138-positive plasma cells in *Nfkb2*^−/−^ lamina propria, but the heterozygous mice showed similar numbers to the wild-type controls. CD138-positive cell numbers also showed a clear *RelB* gene dose dependency, where the plasma cell numbers were reduced in *RelB*^+/−^ and completely absent in *RelB*^−/−^ sections compared with wild type (Fig. 4*D*).

### *Nfkb2-* and *RelB*-deficient Mice Show Different Responses to LPS-Induced Small Intestinal Apoptosis

**Figure 5.**
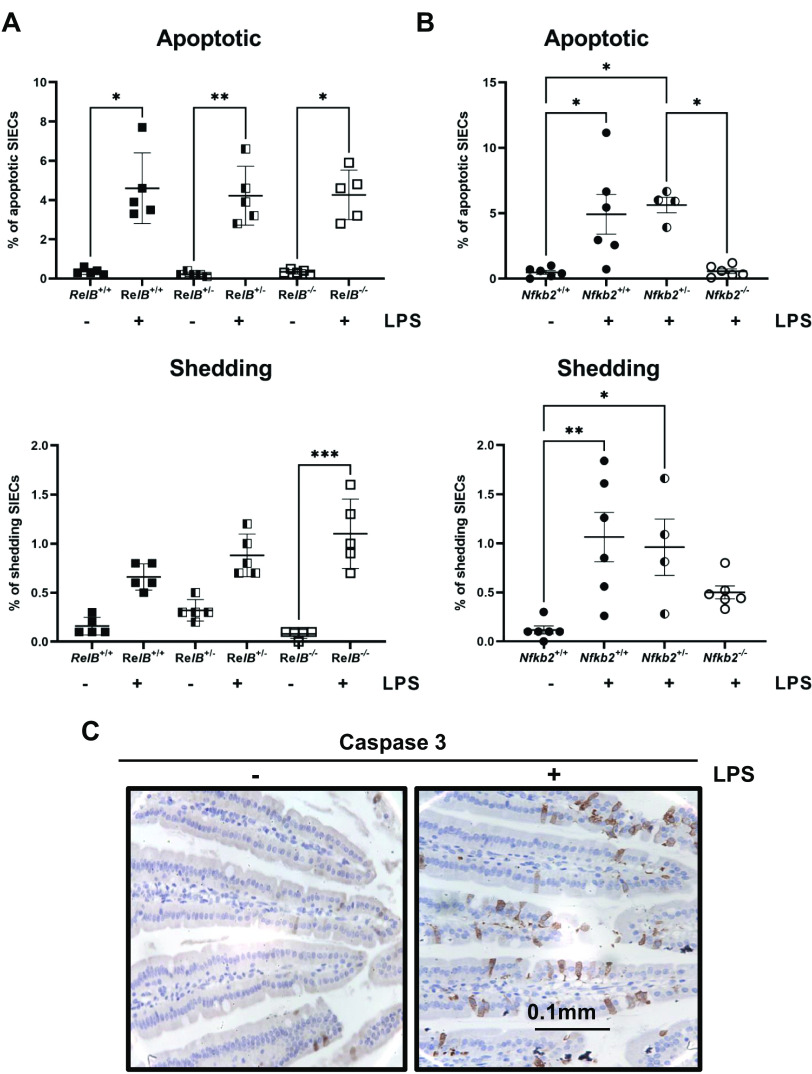
*RelB*-deficient and *Nfkb2* heterozygous mice are sensitive to LPS-induced small intestinal apoptosis in vivo. *A*: quantification of apoptotic and shedding intestinal epithelial cells (ICEs) in *RelB*^+/+^ (black squares), *RelB*^+/−^ (black and white squares), and *RelB*^−/−^ (open squares), small intestinal sections labelled for active caspase-3, 1.5 h after 0.125 mg/kg LPS injection (*n* = 5 mice). *B*: quantification of apoptotic and shedding IECs in *Nfkb2*^+/+^ (black circles), *Nfkb2*^+/−^ (black and white circles), and *Nfkb2*^−/−^ (open circles), small intestinal sections labelled for active caspase-3, 1.5 h after 0.125 mg/kg LPS injection (*n* = 4–6 mice). *C*: IHC (×40) for active caspase 3 in small intestinal sections from C57BL/6 mice without treatment (−) or 1.5 h post 0.125 mg/kg LPS injection (+). Kruskal–Wallis multiple-comparison test was applied. *P < 0.05, **P < 0.01, ***P < 0.001. Scale bars represent 100 μm. LPS, lipopolysaccharide.

In previous studies, we showed that *Nfkb2*^−/−^ mice are resistant to low-dose (0.125 mg/kg body wt) LPS-induced small intestinal apoptosis and that there was undetectable active caspase 3 activity in proximal small intestinal epithelial cells 1.5 h after administration of this stimulus (27). Therefore, we wanted to examine whether gut plasma cells, whose numbers appear to be regulated by the expression of both NF-κB2 and RelB, are involved in this protective mechanism (Fig. 4*D*). For this purpose, the same experiment was performed by injecting low-dose LPS (0.125 mg/kg body wt) and euthanizing the mice 1.5 h later. *Nfkb2*^−/−^ and *RelB*^−/−^ mice were compared with their wild-type and heterozygous littermate controls. Results showed that both *RelB*^−/−^ and *RelB*^+/−^ mice were sensitive to LPS-induced small intestinal epithelial apoptosis to the same extent as their wild-type littermate controls, having ∼4.5% apoptotic and 1% shedding cells in both cases (Fig. 5*A*). Similarly, there was no difference between *Nfkb2^+/−^* and *Nfkb2^+/+^* mice, with both being sensitive to apoptosis and showing 5% apoptotic and 1% shedding cells (Fig. 5*B*). Instead, the *Nfkb2*^−/−^ mice were resistant to LPS-induced caspase 3 activation, as reported previously (27). The small intestine was dissected and fixed in 4% paraformaldehyde. Immunohistochemistry was performed on tissue sections to identify active caspase 3-positive epithelial cells, and these were further categorized as being either apoptotic or apoptotic with shedding morphology (Fig. 5*C*). These data confirm our previous observations in wild-type mice that showed sensitivity to LPS-induced apoptosis (27). Moreover, although the p52 and RelB NF-κB subunits seem to regulate the numbers of CD138-positive plasma cells in the gut, they do not appear to be involved in the responses directing intestinal epithelial apoptosis.

## DISCUSSION

In many experimental models of gastrointestinal diseases, deficiency of the *Nfkb2* gene presents a distinct phenotype compared with phenotypes observed following deficiency of other NF-κB subunits, such as *Nfkb1* or *cRel* (25–27). To identify basal differences in the murine small intestine in the absence of the *Nfkb2* gene, we took a high-throughput approach and applied RNA sequencing on mucosal scrapes isolated from wild-type and *Nfkb2*^−/−^ mice. A striking B cell deficiency was identified, with more than 50% of the downregulated genes being B cell-specific. This signature was also validated using a proteomics approach. Among the downregulated genes, molecules that are important for B cell homing in Peyer’s patches, such as *Cd22* and *St6gal1,* were identified. CD22 is a B cell-associated lectin-homing receptor and its extracellular domain binds to α 2,6-linked sialic acid ligands linked to galactose, a modification that is catalyzed by the enzyme α-2,6 sialyltransferase 1 (ST6Gal1) (46, 49). We investigated the expression of St6Gal1 and showed a significant reduction at the mRNA level and absence of the protein in the lamina propria of *Nfkb2*^−/−^ mice by immunohistochemistry. These findings agree with previous studies that have shown that the absence of the alternative NF-κB pathway is associated with defects in mucosa-associated lymphoid tissue development. Defective organogenesis of Peyer’s patches has been reported previously in *Nfkb1*-, *Nfkb2*-, and *Bcl3-*deficient mice (50). In that study, Peyer’s patches could not be identified macroscopically in the *Nfkb2*^−/−^ strain, which also showed B cell defects in this specific lymphoid tissue. Studies using global *Nik*^−/−^ mice have also shown that loss of NIK signaling, and therefore loss of NF-κB2 activation, decreases both intestinal and systemic humoral response due to a defect in B cell germinal center development (24).

To further characterize the St6Gal1-positive population, staining for CD138/Syndecan-1 was performed. CD138/Syndecan-1 is a member of the syndecan family that comprises heparan sulfate proteoglycans (51). CD138 is involved in cell-cell and cell-matrix interactions and is also a marker for terminally differentiated plasma cells (52). We demonstrated that the St6Gal1-expressing cells within lamina propria were likely to be plasma cells. These cells were severely reduced in number in the *Nfkb2*^−/−^ small intestine and completely absent in the same tissue from *RelB*^−/−^ mice. Heterozygosity of the *Nfkb2* gene did not however affect the number of Cd138+ cells, but a gene dose-dependent defect was observed in *RelB*-deficient small intestine. In addition, *Nfkb2*^−/−^ intestinal epithelial cells also showed some Cd138/Syndecan-1 staining, but this was completely abolished in equivalent *RelB*^−/−^ tissue. CD138 staining appeared to be localized at the basolateral surface of intestinal epithelial cells, as has been reported previously (53, 54). One of the functions of CD138 in epithelial cells is to negatively regulate leukocyte adhesion and migration during inflammation (55). Therefore, its absence could potentially explain the significant inflammatory cell infiltration that has been reported in several organs, including the gastrointestinal tract, in *Relb^−/−^* mice (30, 56).

Plasma cells are most recognizable for their extended lifespan as well as their ability to secrete large amounts of antibodies and act as a key component of humoral immunity (57). De Silva et al. (58), using conditional deletion of *relb* and *nfkb2* in germinal center B cells, showed that RelB/NF-κB2 double deficiency, but not individual deletion of these transcription factors, resulted in the collapse of established splenic germinal centers. In contrast, overexpression of p52 in *Nfkb2*^ΔCt/Δct^ mice resulted in lymphocytic infiltration in various tissues (59). McCarthy et al. (60), using a lymphoma-associated NF-κB2 mutant and human multiple myeloma cell lines, showed that p52 regulates the survival and proliferation of plasma cell tumors. Moreover, frameshift mutations and deletions associated with the *NFKB2* gene in humans result in the production of a truncated p100 protein devoid of the COOH-terminal inhibitory domain, and these are associated with multiple myeloma (61). In the gut, plasma cells secrete antibodies, such as IgA and IgM, and play a crucial role in the maintenance of intestinal homeostasis (62). IgA was undetectable in the serum of *Nfkb2^−/−^* mice, as previously reported (15). IgA was also absent in small intestinal tissue lysates from *Nfkb2^−/−^* mice. This finding confirmed our proteomics data in which IgA was the most downregulated protein in small intestinal tissue. This result is possibly due to the significant developmental defect of Peyer’s patches observed specifically in *Nfkb2^−/−^* mice, but not in the *Nfkb1^−/−^* or *Bcl3^−/−^* mice, that have been reported previously (50) and is characterized by a substantially small B lymphocyte number in the gut. Moreover, previous studies on cytokine profiling of human small intestinal biopsy cultures showed that the alternative NF-κB pathway was necessary for plasma cell survival and antibody secretion (63). In contrast, serum IgM concentrations were higher in *Nfkb2^−/−^* than wild-type mice, a phenotype that has also been observed in the original study in which this *Nfkb2^−/−^* mouse was reported (15). Substantially higher levels of IgM could contribute to higher immunity because IgM is the only immunoglobulin that can be expressed without isotype switching and IgM antibodies are the first to be produced in a humoral immune response (64). IgG2a and IgG3 were also detected at higher concentrations in the small intestinal tissue lysates of *Nfkb2*-deficient animals. This could potentially be another protective mechanism based on previous studies which have demonstrated that in mice under homeostatic conditions, a small subset of Gram-negative symbiotic bacteria in the gut are capable of inducing systemic IgG, which confers critical protection against systemic infections by symbiotic bacteria and pathogens through recognition of conserved antigens (65).

We further tested whether *Nfkb2*^+/−^ mice were resistant to LPS-induced intestinal epithelial apoptosis, as we had previously observed in *Nfkb2^−/−^* mice, the homozygous knockout, during an in vivo model involving intraperitoneal LPS injection (27). Our results revealed that *Nfkb2*^+/−^ mice, as well as *RelB*^+/−^ and *RelB^−/−^* mice were equally sensitive to LPS-induced apoptosis and showed similar levels of active caspase 3 signal as their wild-type littermate controls. These results imply that the main subunits of the alternative NF-κB pathway, p52 and RelB, do not always share common downstream targets. They both seem to regulate the development/homing of plasma cells in the gut, but on the other side, p52 is involved in the activation of caspase-3 and signal-induced apoptosis, whereas RelB is not. This is not a surprise though since it is known that *1*) p52 can form active homodimers and various heterodimers with other partners, such as RelA (66), and *2*) the various dimers can activate distinct subsets of target genes (67, 68). Nevertheless, plasma cells have been implicated in the pathogenesis of various gastrointestinal diseases, such as inflammatory bowel disease (69, 70). The human gut mucosa contains IgM-producing plasma cells in numbers much higher than in mice, and it was shown that they help to anchor highly diverse microbial communities to gut mucus, including Firmicutes with putative beneficial functions (71). Moreover, IgM levels have been reported to confer protection against gut infections. Sahputra et al. (72) showed that the IgMi mice that do not secrete IgM have defective responses to *T. muris* infection, supporting the idea that B cells maintain gut homeostasis during chronic *T. muris* infection via an antibody-dependent mechanism. Further studies are now warranted to determine whether perturbed plasma cell regulation as a result of altered alternative pathway NF-κB signaling affects small intestinal and colonic epithelial cell dynamics and gastrointestinal mucosal function at later time points and following different gastrointestinal tract-damaging stimuli under acute and chronic conditions.

In summary, we have used high-throughput approaches to identify the consequences of *Nfkb2* deficiency on small intestinal tissue from naïve mice. Dysregulation of gene and protein expression related to the immune system was identified and a B cell defect was the main transcriptional signature. Further analyses identified a lamina propria defect of CD138+ plasma cells and small intestinal-specific dysregulation of immunoglobulins, such as absence of IgA and highly increased levels of IgM. Tissue-specific dysregulation of these immunoglobulins may potentially confer protection to *Nfkb2*-deficient mice and further studies are necessary to investigate this hypothesis.

## SUPPLEMENTAL DATA

10.6084/m9.figshare.19146497.v1 Supplemental Tables S1 and S2 and Supplemental Figs. S1 and S2: https://doi.org/10.6084/m9.figshare.19146497.v1.

## GRANTS

Transcriptomic analysis was funded by the SysmedIBD project (www.sysmedibd.eu/) with funding support from the European Community Seventh Framework Program (FP7 – Health; 2007–2013) under Grant Agreement ID No. 305564. Proteomics was supported by a tenure-track fellowship from the University of Liverpool (to C.A.D.) and Libyan Embassy PhD studentship funding (to A.H.E. and D.M.P.). N.G. was supported by The Wellcome Trust through the 4-yr PhD program in Molecular & Cellular Physiology at the University of Liverpool (102172/B/13/Z).

## DISCLOSURES

D.M.P. has received consultancy funding from Ipsen, Advanced Accelerator Applications and Mayoly Spindler laboratories and research funding from Trio Medicines, Ltd. None of the other authors has any conflicts of interest, financial or otherwise, to disclose.

## AUTHOR CONTRIBUTIONS

S.P., J.M.W., W.M., C.A.D., and D.M.P. conceived and designed research; S.P., J.T., A.H.E., J.M.W., N.G., F.I.I., M.T.A., and J.R.H.-F. performed experiments; S.P., J.T., A.H.E., J.M.W., R.S.-T., M.T.A., J.R.H.-F., and C.A.D. analyzed data; S.P., J.M.W., J.R.H.-F., W.M., C.A.D., and D.M.P. interpreted results of experiments; S.P., J.T., M.T.A., and J.R.H.-F. prepared figures; S.P. drafted manuscript; S.P., J.T., J.M.W., R.S.-T., J.R.H.-F., J.H.C., C.S.P., W.M., C.A.D., and D.M.P. edited and revised manuscript; S.P., J.T., A.H.E., J.M.W., F.I.I., R.S.-T., M.T.A., J.R.H.-F., J.H.C., C.S.P., W.M., C.A.D., and D.M.P. approved final version of manuscript.
